# Role of Phytochemicals in Treatment of Aging and Cancer: Focus on Mechanism of FOXO3 Activation

**DOI:** 10.3390/antiox13091099

**Published:** 2024-09-11

**Authors:** See-Hyoung Park

**Affiliations:** Department of Biological and Chemical Engineering, Hongik University, Sejong 30016, Republic of Korea; shpark74@hongik.ac.kr; Tel: +82-44-860-2126

**Keywords:** plant extracts, phytochemicals, FOXO3, anti-aging, anti-cancer

## Abstract

There have been many studies reporting that the regular consumption of fruits and vegetables is associated with reduced risks of cancer and age-related chronic diseases. Recent studies have demonstrated that reducing reactive oxygen species and inflammation by phytochemicals derived from natural sources can extend lifespans in a range of model organisms. Phytochemicals derived from fruits and vegetables have been known to display both preventative and suppressive activities against various types of cancer via in vitro and in vivo research by interfering with cellular processes critical for tumor development. The current challenge lies in creating tailored supplements containing specific phytochemicals for individual needs. Achieving this goal requires a deeper understanding of the molecular mechanisms through which phytochemicals affect human health. In this review, we examine recently (from 2010 to 2024) reported plant extracts and phytochemicals with established anti-aging and anti-cancer effects via the activation of FOXO3 transcriptional factor. Additionally, we provide an overview of the cellular and molecular mechanisms by which these molecules exert their anti-aging and anti-cancer effects in specific model systems. Lastly, we discuss the limitations of the current research approach and outline for potential future directions in this field.

## 1. Introduction

Plants and their extracts have been employed for medicinal purposes since ancient times, primarily due to their phytochemical composition. Phytochemicals are naturally existing organic compounds in plants that show promise as agents for treating various diseases with beneficial effects [1]. Phytochemicals can be categorized as either primary or secondary metabolites. Primary metabolites play a crucial role in the basic plant metabolic processes necessary for growth, development, and reproduction. Primary phytochemicals include carbohydrates, lipids, proteins, and nucleic acids. These are the fundamental building blocks of plant cells and are critical for energy production, cell structure, and genetic information. Secondary metabolites are involved in the interaction of plants with their environment, including defense mechanisms against predators, pathogens, and environmental stress. Secondary phytochemicals include terpenoids, phenolic compounds, alkaloids, and sulfur-containing compounds. They often contribute to the flavor, color, and aroma of plants [2,3]. The relationship between phytochemicals and health benefits is a subject of extensive research, and numerous studies have highlighted the positive impact of consuming plant-based foods rich in phytochemicals on human health [2,4]. Thus, conducting in-depth research on the therapeutic potential of phytochemicals holds great promise, since phytochemicals contain diverse components that could collectively act against multifactorial diseases.

Forkhead box O3 (FOXO3) belongs to a family of forkhead transcription factors that are involved in various cellular processes, such as apoptosis, cell cycle regulation, DNA repair, metabolism, and oxidative stress regulation. A family of forkhead transcription factors is comprised of FOXO1, FOXO3, FOXO4, and FOXO6 proteins. All four isoforms exhibit a shared structural motif known as the “forkhead box”. This domain plays a crucial role in binding to promoters for specific genes [5]. FOXO3 expression is influenced by environmental factors, such as insulin/insulin-like growth factor 1 (IGF1) signaling, oxidative stress, and growth factor deprivation. When activated, FOXO3 translocates to the nucleus and binds to specific promoters to regulate the expression of target genes. Some of these genes are related to the regulation of the cell cycle and oxidative stress, such as superoxide dismutase 2 (SOD2), catalase (CAT), growth arrest and DNA-damage-inducible protein 45α (GADD45α), p27, and BCL-2 interacting mediator of cell death (BIM) [6,7,8].

Thus, FOXO3 has been reported as a promising target for therapeutic intervention in a variety of diseases. FOXO3 has been shown to play a key role in vascular aging and homeostasis by modulating inflammation, oxidative stress, senescence, and angiogenesis. For example, FOXO3 can suppress the expression of pro-inflammatory cytokines, such as tumor necrosis factor α (TNFα) and IL6, and induce the expression of anti-inflammatory cytokines, such as IL10 [9,10]. FOXO3 can also protect vascular cells from oxidative stress by inducing the expression of anti-oxidant enzymes, such as SOD2 and CAT [11]. Moreover, FOXO3 can regulate vascular cell senescence by influencing the expression of cell cycle inhibitors, such as p27, and DNA damage response genes, such as GADD45α and ATM [12,13]. In addition, FOXO3 is known to be involved in a variety of diseases, including cancer and diabetes. In cancer, FOXO3 can inhibit tumor growth and metastasis by regulating the expression of genes responsible for cell proliferation, angiogenesis, and invasion [6,14,15]. FOXO3 increases BIM expression, leading to the induction of apoptosis in breast and liver cancer cells [16]. FOXO3 plays an important role in inducing apoptosis in breast and lung cancer cell lines relying on ATM, CHK2, and phosphorylated p53 isoforms following DNA damage [6]. The knockdown of FOXO3 attenuates Polyphyllin I-induced apoptosis in bladder cancer cells [17]. The reduced expression of FOXO3 has often been found in numerous human cancers with impairment of its anti-cancer capability [8].

In this review, we explore the activation of FOXO3 by natural product extracts and phytochemicals, and their regulation of numerous pathways involved in essential cellular processes. Natural product extracts and phytochemicals capable of activating FOXO3 can present the most promising strategy for enhancing human lifespan with improved health. This review will concentrate on the potential anti-aging and anti-cancer effects of natural product extracts and phytochemicals activating FOXO3.

## 2. Involvement of FOXO3 in Aging

Individuals with extended lifespans typically attain improved longevity, despite comparable risks of diseases. This implies that their genetic information contains protective variants, rather than disease-promoting variants [18]. FOXO3 is one of the genes consistently identified to have an association with human longevity in numerous studies. For instance, a recent study found an association between three specific single-nucleotide polymorphisms (SNPs) of FOXO3 and the exceptionally old age of Japanese American men over 95 years [19]. Similarly, another study showed that two FOXO3 SNPs were significantly associated with longevity by confirming in silico simulation results and in vitro luciferase reporter assay results [20]. These SNPs may affect the splicing, stability, or function of FOXO3 mRNA or protein. While there is an association between SNP variations and FOXO3 expression, the complete molecular effects of FOXO3 SNPs on human longevity might have not been fully understood. The expression of FOXO3 in various model organisms through experimental interventions can either extend or shorten their lifespan [21]. For example, a reduction of 25% in the FOXO3 protein expression level was observed in the skeletal muscle of aged mice compared with young mice [22]. Furthermore, an extension of lifespan was observed when the overexpression of FOXO3 was limited to the fat body of *Drosophila melanogaster* (*D. melanogaster*) [23]. FOXO3 activation leading to longevity may involve various molecular mechanisms. As a transcription factor, FOXO3 is central to cellular stress resistance, energy balance, regulation of the cell cycle, and maintenance of stem cell equilibrium. These functions collectively contribute to improved somatic maintenance and an extended lifespan. Thus, FOXO3 emerges as a highly compelling candidate gene with the aim of enhancing healthy aging and possibly extending lifespan in humans.

## 3. Plant Extracts with Anti-Aging Activity

Plant extracts have been identified as potential agents capable of activating FOXO3 linked to increased lifespan. These plant extracts may exert effects on FOXO3 through specific molecular pathways, such as cellular repair and defense mechanisms. Understanding how plant compounds activate FOXO3 provides insights into potential anti-aging strategies. A significant portion of the regulatory pathways of FOXO3 comes from studies conducted in the nematode *Caenorhabditis elegans* (*C. elegans*). In *C. elegans*, daf16 is an ortholog of FOXO family transcription factor genes. Thus, the activation of DAF16 in *C. elegans* is considered equivalent to the activation of mammalian FOXO3. Several of these investigations have discovered that administering plant extracts leads to an increase in lifespan and enhanced resistance to stress. This phenomenon frequently correlates with the up-regulation of DAF16/FOXO3.

*Myrciaria trunciflora* extract activates the DAF16/FOXO3 pathway against various stressors, such as thermal and ultraviolet radiation in *C. elegans* [24]. *Myrciaria trunciflora* extract (250 μg/mL) demonstrated a capability to substantially enhance the mean lifespan of *C. elegans* (about 20%) compared with the control group under pesticide exposure or heat shock. *Holothuria scabra* and *Agrimonia procera* Wallr. extract exhibited anti-oxidant properties and enhanced stress resistance, leading to an extension in the lifespan of *C. elegans* through the involvement of DAF16/FOXO3 [25,26]. *Agrimonia procera* extract (100 μg/mL) effectively enhanced lifespan (up to 12.7%) and improved resistance to thermal stress (up to 22%). The highest anti-aging impact was observed in *C. elegans* treated with *Holothuria scabra* whole-body butanol extract (500 μg/mL), showing enhanced mean lifespans by 8.12% compared with the control. Longevity and stress tolerance were enhanced in *C. elegans* through the insulin/IGF1 signaling pathway and DAF16/FOXO3 by the administration of raspberry (*Rubus idaeus* L.) extract [27]. The mean lifespan of *C. elegans* treated with raspberry extract (20, 40, and 80 mg/mL) exhibited significant increases of 13.6, 22.9, and 29.7%. Leaf extracts from *Glochidion zeylanicum* extended lifespan and enhanced resistance to oxidative stress in *C. elegans* through the DAF16/FOXO3 and SKN1 (mammalian NRF2 ortholog) signaling pathways [28]. *Glochidion zeylanicum* methanol extracts elevated the survival rate in the presence of oxidative stress induced by juglone compared with the control. Specifically, 2.5 μg/mL of *Glochidion zeylanicum* methanol extracts reduced mortality by 48.82% which was comparable to that of 25 μg/mL of epigallocatechin-3-gallate (EGCG, 40.80%). *Moringa oleifera* extract extended lifespan through DAF16/FOXO3 in *C. elegans* [29]. In the presence of 100 µg/mL of *Moringa oleifera* extract, they found a significant 20.4% increase in the lifespan of *C. elegans*. The mean lifespan for untreated *C. elegans* was 14.2 days, while it extended to 17.7 days for *C. elegans* treated with 100 µg/mL of *Moringa oleifera* extract. The essential oil derived from cloves (*Eugenia caryophyllata* Thunb.) displayed anti-oxidant properties and extended lifespan in *C. elegans* through the activation of DAF16/FOXO3 [30]. *C. elegans* subjected to 1 mg/mL of essential oil derived from cloves exhibited the most substantial promotion of longevity, reaching 15.3% compared with the control group. The essential oil derived from cloves demonstrated anti-oxidant capabilities against reactive oxygen species (ROS) by stimulating the expression of SOD3 or GST4. Liangyi Gao formula (1 mg/mL) prolonged lifespan (23.85) and demonstrated anti-aging effects in *C. elegans* by regulating the DAF16/FOXO3 pathway [31]. Liangyi Gao is a traditional Chinese medicine formula, which primarily consists of two ingredients (*Panax ginseng* and *Rehmanniae radix*). The Liangyi Gao formula included *Panax ginseng* and *Rehmanniae radix* in a 1:2 ratio. *Hibiscus sabdariffa* L. extract extended lifespan and provided protection against amyloid-β toxicity in *C. elegans*, with the participation of DAF16/FOXO3 and SKN1/NRF2 [32]. The lifespan of *C. elegans* was positively influenced by treatment with *Hibiscus sabdariffa* L. extract in a dose-dependent manner, with 24% and 6% increases in median lifespan observed after incubation with 1.0 and 0.5 mg/mL *Hibiscus sabdariffa* L. extract. By employing a transgenic *C. elegans* model that was transfected with a temperature-induced expression system of the human amyloid-β peptide, they demonstrated that *Hibiscus sabdariffa* L. extract resulted in a delayed onset of paralysis by 2 h compared with the control. Although most research has been conducted on *C. elegans*, several studies have reported on lifespan extension by natural product extract for FOXO3 activation using *D. melanogaster*. Korean mistletoe (*Viscum album* var. *coloratum*) extract prolonged the lifespan of *D. melanogaster* through the activation of FOXO induced by *D. melanogaster* Sir2 (dSir2) [33]. *Viscum album* var. *coloratum* extract supplementation (25 μg/mL) resulted in a 5.45% increase in the mean lifespan of male *D. melanogaster* and a significant 21.02% increase in female *D. melanogaster*. *Viscum album* var. *coloratum* extract did not extend the lifespan of male *D. melanogaster* and increased the mean lifespan of female *D. melanogaster* by 5.2% in a dSir2 null mutant. These findings suggest that the lifespan extension induced by *Viscum album* var. *coloratum* extract supplementation is mediated by sirtuin, which is a well-known upstream deacetylase, to promote the transcriptional activity of FOXO3. In fact, they indicated that the activation of FOXO3 transcription factor by *Viscum album* var. *coloratum* extract was dependent upon dSir2.

**Figure 1:**
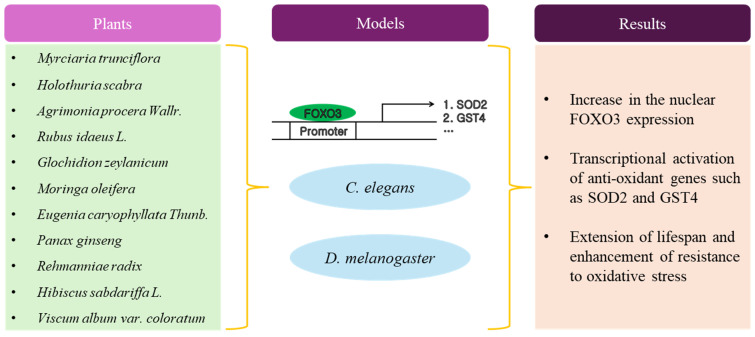
Schematic diagram of anti-aging activity by plant extracts.

As shown in Table 1 and Figure 1, most studies adopted the *C. elegans* model to explain the relationship between FOXO3 and longevity by the administration of plant extract, but there have been several experimental studies using *D. melanogaster* [34,35,36] or yeast [37,38]. *C. elegans* is advantageous for studying longevity due to its short lifespan, enabling rapid observation of the entire aging process. *C. elegans*’ genetic tractability allows researchers to easily manipulate and study aging-related genes and pathways [39,40]. Many aging-related genes in *C. elegans* are evolutionarily conserved, offering insights applicable to higher organisms. Its transparent body facilitates non-invasive, real-time visualization of internal processes. Furthermore, the ease of culture and availability of tools make *C. elegans* a cost-effective and efficient model for aging research [41]. Although not all studies were reviewed in this paper, generally, researchers treated *C. elegans* with plant extract doses ranging from about 10 ug/mL to 100 mg/mL. The mean lifespan of *C. elegans* was increased by up to about 30% compared with the control. Regarding the working mechanism of the plant extract for longevity, researchers posited that the plant extract could increase resistance against environmental stress such as heat, ultraviolet, and chemical-mediated oxidative stress.

While plant extracts have been used for medicinal purposes for centuries and continue to be a valuable source of pharmaceutical compounds, functional food ingredients, and cosmeceuticals, there are some limitations associated with their use [42,43,44]. The composition of plant extracts can vary, leading to challenges in achieving consistent and standardized formulations for medicinal or nutraceutical use. Some bioactive compounds in plant extracts may have poor bioavailability, impacting their absorption and effectiveness in the body. Also, toxic components from plant extracts or interactions with other medications may pose safety risks, requiring thorough testing and monitoring. The exact mechanisms of action for many plant-derived compounds may not be fully understood, hindering precise predictions of their medicinal effects. Thus, further research should be needed to explore the full potential of plant extracts targeting FOXO3 for promoting longevity.

## 4. Phytochemicals with Anti-Aging Activity

Despite the limitations of plant extracts, many studies have explored ways to address these challenges and harness the therapeutic potential of plant extracts in a more systematic and reliable manner. Advances in technology and understanding of plant chemistry contribute to improving the development and utilization of plant-derived phytochemicals [45]. Phytochemicals have shown promising potential as anti-aging agents by influencing the activation of FOXO3. For example, resveratrol found in red grapes and berries has been linked to the activation of FOXO3, promoting cellular health and longevity [46,47,48]. Additionally, flavonoids, present in fruits and vegetables, may stimulate FOXO3, contributing to cellular repair mechanisms [49,50].

Dihydromyricetin extends lifespan and triggers the activation of FOXO3 in *D. melanogaster* [51]. In comparison with *D. melanogaster* exposed to control food with 0 μM dihydromyricetin, those fed with 40 μM dihydromyricetin exhibited elevated median lifespans by 16.07%. Dihydromyricetin promoted the localization of FOXO3 in the nucleus through decreasing the phosphorylated AKT (pAKT) level in *D. melanogaster*. Kaempferol and fisetin diminished cellular ROS levels, decreased vulnerability to oxidative stress, and promoted the nuclear translocation of the transcription factor DAF16/FOXO3 in *C. elegans* [52]. The mean lifespans of *C. elegans* treated with 100 μM kaempferol and fisetin increased by approximately 10% and 6%, respectively, under thermal stress. Chlorogenic acid extended lifespan via the activation of SKN1/NRF2 and DAF16/FOXO3 in *C. elegans* [53]. A dose of 20 μM of chlorogenic acid extended the mean lifespan of *C. elegans* (DAF16/FOXO3 mutant) rescued by DAF16a and DAF16f by 24% and 9%, respectively. This implied that both isoforms contributed to the lifespan-extending effects of chlorogenic acid and DAF16a played a more significant role than DAF16f. Unexpectedly, chlorogenic acid prompted the cytoplasmic retention of DAF16a and nuclear retention of DAF16f, indicating that chlorogenic acid inhibited DAF16a, but activated DAF16f. Further studies are required to clarify the precise mechanism by which chlorogenic acid causes DAF16/FOXO3 isomer localization in cells. Sulforaphane enhanced the longevity of *C. elegans* through the DAF16/FOXO3 [54]. While the mean lifespan of the control *C. elegans* was 16 days, exposure to concentrations of 100, 200, and 400 μM of sulforaphane resulted in significant increases in survival of 18.2%, 13.31%, and 15.46%, respectively. Sulforaphane suppressed insulin/IGF1 signaling along with its downstream targets AGE1, AKT1, and AKT2. This led to an elevated nuclear translocation of DAF16/FOXO3. Consequently, it led to the up-regulation of target genes, such as SOD3, MTL1, and GST4, known for enhancing stress resistance. Anti-aging effects of phlorizin in *C. elegans* are achieved through the activation of DAF16/FOXO3 [55]. The mean lifespan increased significantly from 17.5 days (untreated control group) to 20.6 days (10 μM phlorizin-treated group, 18% increase). Additionally, the maximum lifespan extended from 23 days (untreated control group) to 27 days (10 μM phlorizin-treated group). Phlorizin caused the up-regulation of HSP16.2 and SOD3 expression, along with the nuclear translocation of DAF16/FOXO3 in *C. elegans*. Caffeic acid phenethyl ester augmented stress resistance and prolonged lifespan in *C. elegans* by influencing the DAF16/FOXO3 signaling pathway [56]. Caffeic acid phenethyl ester (100 μM) reduced thermal stress-mediated ROS levels by approximately 50%. Caffeic acid phenethyl ester induced the nuclear translocation of DAF16/FOXO3, leading to a 9% increase in median lifespan and 17% increase in maximum lifespan. These prolonged lifespan effects of caffeic acid phenethyl ester were dependent on DAF16/FOXO3, which was demonstrated in experiments using a DAF16/FOXO3 loss-of-function mutant strain. The colonic metabolites of ferulic acid promoted longevity and increased stress resistance in *C. elegans* through the activation of DAF16/FOXO3 [57]. Under normal conditions in *C. elegans*, 0.5 mM 3-(3,4-dihydroxyphenyl) propionic acid, 2.5 mM 3-(3-hydroxyphenyl) propionic acid, and 2.5 mM 3-phenyl propionic acid extended mean lifespans by 11.2, 13.0, and 10.6%, respectively, whereas ferulic acid did not exert similar effects. Additionally, treatments with these metabolites of ferulic acid enhanced stress tolerance against heat and UV irradiation and induced the translocation of DAF16/FOXO3. Life span extension in *C. elegans* was facilitated by methylated derivatives of myricetin depending on DAF16/FOXO3 [58]. Similar to the effect of myricetin on prolonging lifespan (48.2%), the methylated myricetin derivatives laricitrin, syringetin, and myricetin trimethyl ether (100 μM) significantly increased the lifespan of *C. elegans* by 35.7, 54.5, and 53.6%, respectively. Unlike myricetin, the methylated compounds significantly improved resistance to thermal stress. Additionally, the derivatives induced a more pronounced nuclear localization of DAF16/FOXO3. The lifespan-promoting effects of betalains in *C. elegans* were mediated through DAF16/FOXO3 and SKN1/NRF2 [59]. Betalains are a group of pigments found in some plants and are responsible for the vibrant red, purple, and yellow colors observed in plants. In this study, 10 μM indoline carboxylic acid-betacyanin, 25 μM phenylalanine-betaxanthin, and 100 μM dopaxanthin exhibited remarkable in vivo anti-oxidant and anti-aging properties by extending the lifespan of *C. elegans* by up to 16.55, 12.92 and 20.52% respectively. The microarray results coupled with biological validation using various mutant strains demonstrated that the observed extension of lifespan was attributed to the activation of transcription factors DAF16/FOXO3 and SKN1/NRF2 responsible for the transcription of heat shock protein genes. Damaurone D exerted positive effects on lifespan, making it a valuable natural compound from plants for developing nutraceutical formulations addressing aging and age-related diseases [60]. Damaurone D increased the lifespan of *C. elegans* in a dose-dependent manner. The average lifespan was increased by 7.49, 12.60, and 16.70% when exposed to 5, 10, and 15 μM of damaurone D, respectively. The ability to enhance longevity was attributed to resistance against thermal and osmotic stress and the capabilities and up-regulation of the expressions of stress-response proteins, such as SOD3 and HSP16.2, regulated by DAF16/FOXO3. Tectochrysin enhanced stress resistance and prolonged the lifespan of *C. elegans* through DAF16/FOXO3 [61]. Treatment with 200 μM of tectochrysin showed the largest lifespan extension by 21.0%. The CL4176 strain is characterized by the presence of a transgene that encodes the human amyloid-β peptide (Aβ1-42), making it a genetic model for Alzheimer’s disease (AD). The administration of tectochrysin resulted in a substantial postponement of body paralysis onset in the CL4176 strain that is characterized by the presence of a transgene that encodes amyloid-β (Aβ1-42) and prolonged the lifespan of the CL4176 strain by 14.8%. Syringaresinol has been identified as a compound that slows down cellular senescence by triggering the FOXO3-dependent activation of SIRT1 and modulation of insulin/IGF1 signaling [62]. Syringaresinol induced metabolic alterations similar to those seen in dietary restriction. The administration of 100 and 500 nM syringaresinol led to notable and dose-dependent increases in lifespan of 14 and 41% in *C. elegans*. In addition, 500 nM syringaresinol prolonged the lifespan of *D. melanogaster* by 10.52% (males) and 13.67% (females). The mimetic effects of dietary restriction were facilitated through the FOXO3-dependent activation of SIRT1 and modulation of insulin/IGF1 signaling. The extension of lifespan in *C. elegans* exposed to oxidative stress-inducing chemicals was extended by 100 μM quercetin by 15% [63]. Another study examined the in vivo outcomes of extended administration of low doses of quercetin under conditions simulating natural aging. Fourteen-month-old male C57BL/6J mice were orally administered quercetin on a weekly basis at a concentration of 0.125 mg/kg body weight. Following an eight-month treatment period, mice receiving quercetin exhibited a reduction in hair loss, alongside regular food intake, body weight, blood glucose levels, and bone mineral density [64]. However, low-dose quercetin treatment did not extend their lifespan up to 31 months.

**Figure 2:**
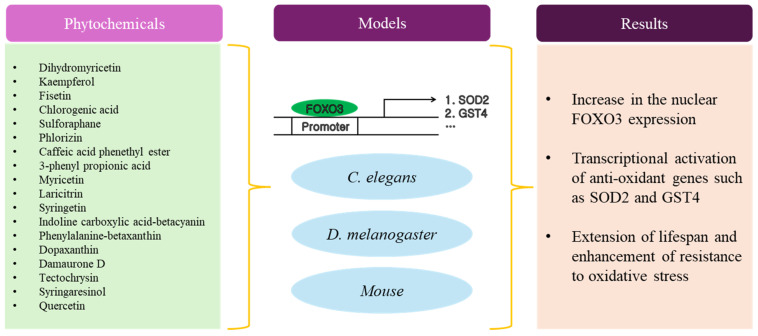
Schematic diagram of anti-aging activity of phytochemicals.

As with research on the anti-aging effect of plant extracts, many studies have adopted *C. elegans* and *D. melanogaster* as a model system for investigating the anti-aging effect of phytochemicals (Table 2 and Figure 2). *C. elegans* were exposed to phytochemicals within a dosage range of approximately 10 nM to 2.5 mM. The average lifespan of *C. elegans* exhibited approximately 10 to 50% augmentation compared with the control group. Regarding the mechanism by which phytochemicals promoted longevity, there have been efforts to demonstrate its ability to enhance resistance against environmental stressors through the induction of HSP16.2 and SOD3. In addition, several studies showed that the anti-aging effect of phytochemicals was associated with both DAF16/FOXO3 and SKN1/NRF2. Most phytochemicals were isolated from plants and had a poly phenolic structure, such as flavonoids. A few studies used the derivative of the original phytochemicals to compare their anti-aging effects.

The lack of a thorough understanding of the mechanisms of action for many phytochemicals may hinder precise predictions of their medicinal effects. Researchers could demonstrate a more detailed working mechanism by which phytochemicals exert anti-aging effects in specific model systems. As with research about plant extracts, there was variability in the treatment dose and time of phytochemicals in each study, which could lead to inconsistent therapeutic effects and difficulties in ensuring reliable and reproducible doses. Addressing these challenges is crucial for the successful development and use of phytochemicals in medicine.

## 5. Involvement of FOXO3 in Cancer

Many studies suggest that the dysregulation of FOXO3 activity is associated with the development and progression of various types of cancer in humans [65,66,67,68,69,70]. Therefore, understanding the mechanisms by which FOXO3 exerts its anti-cancer effects may have implications for the development of targeted therapies in cancer treatment. In addition, it is important to note that the role of FOXO3 in cancer is complex and context-dependent, as its activity can be influenced by various factors in different cellular environments. FOXO3 regulates the expression of various genes involved in apoptosis and cell cycle arrest, such as BIM, Fas ligand (FasL), TNF-related apoptosis-inducing ligand (TRAIL), p53 upregulated modulator of apoptosis (PUMA), p21, p27, and GADD45α [71]. It is inhibited by oncogenic kinases, which are proteins that promote cancer cell growth [72,73,74]. Oncogenic kinases can phosphorylate FOXO3, which leads to ubiquitin-mediated degradation and inactivation of FOXO3. This prevents FOXO3 from binding to DNA and regulating the expression of genes that promote cell cycle arrest and apoptosis. As a result, oncogenic kinases can contribute to the development and progression of cancer. There are a number of different oncogenic kinases that can inhibit FOXO3. Some of the most common include AKT, ERK, and IKKβ [75,76,77]. These kinases are activated by a variety of factors, including growth factors, oncogenes, and environmental toxins. FOXO3 is a promising target for cancer therapy. Drugs that inhibit oncogenic kinases can prevent them from phosphorylating FOXO3 and activating it. This can lead to the death of cancer cells and the inhibition of tumor growth. For instance, it was found that the pharmacological inhibition of AKT and ERK promoted the activation of FOXO3, leading to apoptosis in several types of cancer [78,79,80,81]. These studies suggest that targeting FOXO3 or oncogenic kinases may be a promising strategy for cancer therapy.

## 6. Plant Extracts with Anti-Cancer Activity

*Houttuynia cordata* Thunb extract enhanced the activation of FOXO3, leading to apoptosis in HepG2 hepatocellular carcinoma cells [82]. The exposure of HepG2 cells to 10 µg/mL *Houttuynia cordata* Thunb extract resulted in a significant increase in late apoptosis. The percentage of cells undergoing apoptosis rose from 25.7% in untreated cells to 86.6% in those treated with the extract. They pinpointed HIF1A, FOXO3, and MEF2A as apoptotic triggers activated by *Houttuynia cordata* Thunb extract in HepG2 cells. *Allium Roseum* L. extract displayed strong inhibitory effects on the viability of chronic myeloid leukemia K562 cells by suppressing BCR-ABL, PI3K/AKT, and ERK kinases, as well as reducing VEGF secretion [83]. The most potent inhibition rate of cell proliferation by *Allium Roseum* L. extract was 87% at a concentration of 500 µg/mL for 72 h. *Allium Roseum* L. extract disrupted the normal progression of the cell cycle and triggered apoptosis in K562 cells. The inactivation of AKT kinase by *Allium Roseum* L. extract resulted in the activation of FOXO3 transcription factor. This activation, in turn, increased the expression of pro-apoptotic effectors regulated by FOXO3, such as BIM and BAX, as well as the cell cycle inhibitor p27. *Morus alba* root extract generated ROS to trigger apoptosis through the FOXO3-dependent pathway in B103 rat neuroblastoma cells [84]. *Morus alba* root extract (10 μg/mL) decreased cell viability to around 60% compared with untreated controls in B103 cells. However, *Morus alba* root extract showed comparatively less cytotoxicity in Rat-2, normal rat fibroblast cells. They revealed that *Morus alba* root extract treatment led to the accumulation of ROS and the depolarization of mitochondrial membrane potential in B103 cells. Additionally, *Morus alba* root extract in B103 cells resulted in DNA damage and apoptosis. Western blotting analysis showed that the phosphorylated AKT expression level was decreased by *Morus alba* root extract, which suggested a decline in cellular proliferation and transcription concurrent with apoptosis and was supported by increased FOXO3 and BIM expression. *Chelidonium majus* extract triggered apoptosis in SKOV3, OVCAR3, and MDAH2774 human ovarian cancer cells by increasing FOXO3 expression [85]. At 24 h, the IC50 value for *Chelidonium majus* extract was established at 200 μg/mL, indicating that it suppressed approximately 50% of the growth of the three cell lines. But the viability of human mesenchymal stem cells (hMSCs) remained at or above 60%, even when exposed to concentrations of up to 500 μg/mL of *Chelidonium majus* extract for 48 h. The expression of pro-apoptotic downstream proteins of FOXO3, such as cleaved caspase3 and BAX, was increased, whereas the expression of anti-apoptotic BCL2 was decreased by *Chelidonium majus* extract in SKOV3 cells in a dose-dependent manner. *Fagonia cretica* (recently corrected as *Fagonia indica*) extract induced cell cycle arrest and apoptosis in MCF7 and MDA-MB-231 breast cancer cells through the expression of FOXO3 [86]. The extract showed a stronger cytotoxic effect on MCF7 cells (IC25 = 0.43 mg/mL) compared with MDA-MB-231 cells (IC25 = 1.01 mg/mL) at 24 h. Moreover, it was observed that, even after 72 h, the extract treatment at a concentration of 2 mg/mL caused only a 20% reduction in normal human mammary epithelial cell (HMEpC) viability. This suggests that the extract exhibits greater cytotoxic activity against human breast cancer cell lines. Treatment with the extract increased FOXO3 expression in both MCF7 and MDA-MB-231 cells. Additionally, FOXO3 siRNA resulted in a reduction in the extract-induced cytotoxicity in both cell lines. Flavonoids and rutin extracted from *Hammada scoparia* eliminated both adherent and chemo-resistant leukemia cells [87]. U937, HL60, and KG1 leukemia cell lines exhibited increased susceptibility to treatment with the aqueous *Hammada scoparia* extract when grown in adherent conditions. The viability of cells was reduced to about 50% by treatment with the aqueous *Hammada scoparia* extract (66 μg/mL) for 24 h. Interestingly, the rutin-rich fraction of *Hammada scoparia* extract triggered apoptosis specifically in adherent U937 leukemic cells through the phosphorylation of FOXO3 at Thr32. The apoptosis effect of *Aegiceras corniculatum* leaf extract on human colon cancer cells was induced through the activation of the FOXO3 signaling pathway [88]. The IC50 values of *Aegiceras corniculatum* leaf extract were 34.01 and 37.90 μg/mL for HT-29 and SW480 colon cancer cells. The anti-cancer mechanism of *Aegiceras corniculatum* leaf extract involved the activation of FOXO3 activating the cell cycle kinase inhibitor p21 and pro-apoptotic BIM. In addition, unexpectedly, *Aegiceras corniculatum* leaf extract increased the phosphorylation of FOXO3 at Ser253. *Anagallis arvensis* extract exhibited anti-cancer and radio-sensitizing effects on breast cancer cells by upregulating the expression of FOXO3 [89]. Data on the anti-cancer effect of *Anagallis arvensis* extract on MCF7 and MDA-MB-231 cells showed IC50 values of 33.73 and 48.24 µg/mL, respectively. However, the IC50 values of *Anagallis arvensis* extract did not exhibit toxicity to normal cells. The protein expression of FOXO3 and γH2AX was significantly elevated in MCF7 and MDA-MB-231 cells by *Anagallis arvensis* extract treatment, which was related to DNA-damaging mediated apoptosis in cells.

**Figure 3:**
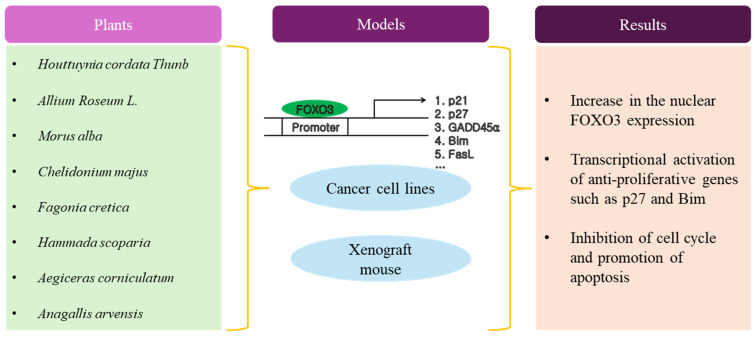
Schematic diagram of anti-cancer activity by plant extracts.

As shown in Table 3 and Figure 3, most studies adopted the in vitro human cancer cell line as a model system to examine the anti-cancer activity of natural plant extracts. While cancer cell lines offer advantages such as availability, uniformity, and cost-effectiveness for studying specific treatments, researchers must also consider their limitations, including the lack of tumor microenvironment representation, genetic stability issues, limited representativeness of patient tumors, and the potential for misinterpretation of results. Integrating findings from cell line studies with data from other model systems and clinical trials can help to mitigate these limitations and enhance the reliability of preclinical research. Generally, researchers treated cancer cells with plant extract doses ranging from about 10 to 1000 ug/mL, leading to at most 90% cytotoxicity in cancer cells for 72 h. As far as the working mechanism of the plant extract for anti-cancer activity, researchers demonstrated that plant extract induced apoptosis in cancer cells by the activation of FOXO3 responsible for the up-regulation of pro-apoptotic BIM and BAX, as well as cell cycle inhibitors.

While plant extracts offer potential benefits as a treatment option for cancer, including their natural origin, rich bioactive compound content, and potential for low toxicity, they also present challenges such as a lack of standardization, limited clinical evidence, potential for interactions and side effects, and regulatory issues. Integrating plant-based therapies into comprehensive cancer care requires careful consideration of these factors and collaboration between traditional medicine practitioners and modern healthcare providers.

## 7. Phytochemicals with Anti-Cancer Activity

Phytochemicals, which are bioactive compounds found in plants, hold significant potential as anti-cancer agents through various mechanisms. There have been various kinds of phytochemicals that have the ability to activate FOXO3 [90,91,92,93,94]. Some examples include curcumin in turmeric, quercetin in apple, resveratrol in red grapes, EGCG in green tea, sulforaphane in broccoli sprouts, and lycopene in tomatoes. Many phytochemicals exhibit anti-oxidant properties, which help to neutralize harmful free radicals in the body. Free radicals can damage DNA and other cellular components, contributing to cancer development [1,95,96]. By scavenging free radicals, phytochemicals can help to prevent oxidative stress and reduce the risk of cancer. Chronic inflammation is associated with the development of various cancers [3,97,98]. Phytochemicals such as flavonoids, polyphenols, and carotenoids possess anti-inflammatory properties, which can inhibit inflammatory pathways implicated in cancer initiation and progression. Apoptosis, or programmed cell death, is a natural process that helps to regulate cell growth and eliminate damaged or abnormal cells, including cancerous cells. Some phytochemicals have been shown to induce apoptosis specifically in cancer cells, triggering their death while sparing healthy cells [99,100,101,102,103,104,105,106,107,108,109,110,111]. Angiogenesis, the formation of new blood vessels, is essential for tumor growth and metastasis. Certain phytochemicals can inhibit angiogenesis by targeting the signaling pathways involved in blood vessel formation, thereby limiting the blood supply to tumors and hindering their progression [99,112,113].

Demethylzeylasteral exhibited anti-cancer activity against non-small-cell lung cancer in vitro and in vivo through the activation of FOXO3 and p53 [114]. Demethylzeylasteral effectively inhibited the growth of H460, H1975, and PC-9 cells, with IC50 values of 8.46, 6.97, and 8.68 µM, respectively. However, demethylzeylasteral showed lower cytotoxicity toward BEAS-2B human normal lung epithelial cells. Demethylzeylasteral decreased the phosphorylation level of AKT at Ser473 and FOXO3 at Thr32, which was associated with the increased expression of p21 and BAX as downstream targets of FOXO3. To assess the in vivo anti-cancer activity of demethylzeylasteral, the PC-9 cell xenograft model was utilized. The results showed that demethylzeylasteral inhibited tumor growth, with the high-dose (5 mg/kg) group exhibiting a stronger anti-tumor effect than the low-dose (2.5 mg/kg) group.

Pinostrobin exhibited anti-leukemic effects through the nuclear localization of FOXO3 in acute myeloid leukemia cells and zebrafish xenografts [115]. The IC50 values of pinostrobin in HL-60, U-937, THP-1, MV4-11, and MOLM-13 cells were 129.9, 919.7, 1458.0, 35.8, and 53.2 μM, respectively. These findings indicated that pinostrobin had significant cytotoxic effects on human acute myeloid leukemia cells, with its anti-leukemia efficacy following the trend of MV4-11 > MOLM-13 > HL-60 > U-937 > THP-1. To assess the effect of pinostrobin on cancer cell growth in zebrafish xenografts, fluorescent dye-labeled MV4-11 cells were microinjected into zebrafish embryos and then treated with pinostrobin (15 and 30 μM) for 48 h. The number of fluorescent MV4-11 cells was significantly reduced in zebrafish xenografts treated with pinostrobin. Pinostrobin (30 and 60 μM) significantly increased both mRNA levels and the protein expression of FOXO3 in MV4-11 cells. Pinostrobin treatment significantly increased nuclear FOXO3 protein levels while decreasing the cytosolic FOXO3 levels. Butein inhibited cell growth in ovarian cancer by regulating the IL6/STAT3/FOXO3 pathway [116]. To investigate the effects of butein on ovarian cancer in vitro, A2780 and SKOV3 cell lines were used. Butein inhibited cell viability with IC50 values of 64.7 and 175.3 μM, respectively. The wound-healing assay showed that butein decreased cell migration at 24 and 48 h in a dose-dependent manner. Cell infiltration was also similarly reduced, as observed in the Matrigel cell invasion assay. Additionally, butein induced cell cycle arrest and apoptosis in these cells. Butein inhibited STAT3 phosphorylation and promoted the nuclear accumulation of FOXO3 by blocking IL6 signaling, leading to the up-regulation of p27. EGCG suppressed ovarian cancer cell growth and induced apoptosis by activating FOXO3 [117]. Treatment with EGCG inhibited cell growth in both A2780 and SKOV3 cells in a dose-dependent manner. The viability of cells treated with 20 nM was about 50% in both cells. EGCG treatment increased FOXO3 expression but decreased c-Myc expression in both cells.

Oleanolic acid caused the apoptosis of HCT116 colon cancer cells by activating the p38 MAPK/FOXO3 pathway [118]. After 72 h of exposure, cell viability was reduced by oleanolic acid in a dose-dependent manner, with an IC50 value of 29.3 μM. Western blot analysis results showed that oleanolic acid increased the expression of FOXO3. Immunofluorescence analysis demonstrated the intracellular localization of FOXO3 in HCT116 cells. And interestingly, the expression of phosphorylated FOXO3 at Ser294 was also increased by oleanolic acid treatment. FOXO3 is known to be phosphorylated at Ser294 by JNK, p38, and ERK MAPK [30,31]. They demonstrated that oleanolic acid promoted the accumulation of phosphorylated FOXO3 at Ser294 in the nucleus by activation of p38 MAPK. Avenanthramide C triggered cellular senescence in colon cancer cells by inhibiting the β-catenin-mediated transcription of miR-183/96/182 cluster, which was shown to be associated with the up-regulation of FOXO3 [119]. The MTT assay results indicated that treatment with avenanthramide C reduced the viability of SW620, SW480, HCT-8, and HCT116 cells in a dose-dependent manner at 24 h. The IC50 values were 153.02, 161.47, 105.23, and 112.69 μM, respectively. Avenanthramide C (25 μM) increased the expression of senescence markers, such as p16, p21, and p27. Mechanistically, they showed that avenanthramide C suppressed the expression of the miR-183/96/182 cluster by reducing β-catenin, leading to cellular senescence in colon cancer cells. Furthermore, consistent with the fact that the miR-183/96/182 cluster directly targets SMAD4 and FOXO3, they observed a significant increase in the levels of SMAD4 and FOXO3 following avenanthramide C treatment in colon cancer cells.

Eugenol triggered apoptosis in breast cancer cells by regulating the AKT/FOXO3 pathway [120]. At concentrations of 5, 10, and 20 μM, the viability of MDA-MB-231 cells was significantly reduced by 20, 35, and 58%, respectively, after 24 h of incubation, and by 40, 65, and 80%, respectively, after 48 h of incubation. Higher concentrations (40 and 60 μM) led to a drastic inhibition of cell viability by more than 90%. Similarly, for SK-BR-3 cells, concentrations of 5, 10, and 20 μM decreased viability by 15, 30, and 70%, respectively, after 24 h of incubation, and by 32, 72, and 80%, respectively, after 48 h of incubation. The expression levels of FOXO3 mRNA and protein were increased by 4 and 8 μM of eugenol compared with the control, which was associated with increased expression of p27. Interestingly, eugenol also induced AKT mRNA and protein expression. In a recent publication [121,122], the researchers described that FOXO3 could activate class I PI3K catalytic subunit PIK3CA at the transcriptional level, which subsequently phosphorylates and enhances AKT activity. The activated AKT then phosphorylates FOXO1, causing its translocation to the cytoplasm and triggering autophagy. Since most studies demonstrate the anti-cancer activity of compounds by proving the induction of FOXO3 activation through the inhibition of AKT, these results seem to be interesting and unique in that they showed the activation aspect of AKT by eugenol in breast cancer cells.

Crocin induced apoptosis in breast cancer cells by generating ROS, involving the PTEN/AKT/FOXO3 signaling pathways [123]. The IC50 values of crocin for MCF7 and MDA-MB-231 were 3 and 2.7 mM at 24 h, respectively, which seemed to be relatively high. However, under the same condition and at concentrations around the IC50 value, crocin had no significant effect on the viability of human primary normal epithelial breast cells. Crocin triggered ROS production, which was confirmed by fluorescent probe for various types of ROS, including H_2_O_2_, NO, and even superoxide anion. Crocin decreased FOXO3 phosphorylation at Ser253 (AKT-dependent phosphorylation site) and AKT phosphorylation at Thr308 (indicating AKT inactivation). These results suggest that Crocin-induced AKT inactivation prevented FOXO3 phosphorylation. Consequently, FOXO3 translocated to the nucleus and activated the transcription of target genes, such as BIM and PTEN. Resveratrol induced apoptosis in cervical cancer HeLa cells by activating FOXO3 and promoting its nuclear translocation [124]. The apoptotic rates in HeLa cells treated with 0, 40, and 80 μM of resveratrol were 8.6, 13.4, and 20.2%, respectively. The exposure of HeLa cells to resveratrol for 48 h increased the total FOXO3 level and decreased the expression of phosphorylated FOXO3 by inhibiting ERK phosphorylation. Unfortunately, the exact phosphorylation site of FOXO3 was not described in the article. Also, resveratrol significantly enhanced FOXO3 translocation from cytosol to the nucleus, which subsequently promoted BIM expression related to apoptosis in HeLa cells.

Quercetin prompted apoptosis in cervical cancer cells by increasing apoptotic genes, such as FOXO1, FOXO3, and p53 [125]. The IC50 value of quercetin was 100 µM after 24 h of treatment in HeLa cells. However, the lymphocytes isolated from fresh blood exhibited no adverse response to quercetin treatment (1–150 µM for 24 h) and their growth was not inhibited at any of the concentrations tested. FOXO1, FOXO3, and p53 were found to be increased by quercetin. Also, other pro-apoptotic responders, such as BIM and FasL were found to be increased in response to quercetin.

Zheng et al. reported that β-elemene increased the expression of FOXO3, contributing to the induction of insulin-like growth factor-binding protein 1 (IGFBP1) expression in lung cancer [126]. The growth of human lung cancer cells H1957 treated with β-elemene was inhibited compared with the control cells. The IC50 value of β-elemene was observed to be around 30 µM after 48 h of treatment. β-elemene increased the protein expression of FOXO3 in H1975 and A549 cells. Interestingly, β-elemene increased the protein and mRNA levels of IGFBP1, as well as IGFBP1 gene promoter activity. Previous reports indicated that the IGFBP1 promoter region could be bound by FOXOs, which could regulate transcriptional IGFBP1 gene expression [127,128]. They found that silencing FOXO3 reduced the effect of β-elemene on both IGFBP1 protein expression and promoter activity in A549 and H1975 cells, suggesting a strong interaction and potential recruitment of FOXO3 in the transcriptional regulation of IGFBP1. Casticin inhibited the development of small-cell lung cancer H446 cells by activating the AMPK/FOXO3 signaling pathway [129]. Second-generation spheres from H446 cells, which exhibit lung cancer stem-like cell (LCSLC) properties, were utilized for the subsequent experiments. Although the experimental values were not expressed in numbers, sphere formation assay results showed that 3 μM of casticin inhibited colony formation by a reduction rate of about 50% compared with the control. Treatment with casticin led to a notable increase in the phosphorylation levels of AMPK, along with a decrease in FOXO3 phosphorylation (Ser253) in a dose-dependent manner. Yung et al. reported that the activation of AMPK could suppress the growth of cervical cancer cells by modulating the AKT/FOXO3/FOXM1 signaling pathway [130]. According to them, the inhibition of FOXO3 (phosphorylation of Ser253) by AKT can be diminished when AMPK is activated (phosphorylated). Thus, this report suggested that casticin suppressed the in vitro carcinogenesis and cancer stem cell characteristics of H446-derived LCSLCs, potentially through the activation of the AMPK/FOXO3 signaling pathway. Delphinidin triggered apoptosis and autophagy in HER-2-positive breast cancer cells by activating the AMPK/FOXO3 pathway [131]. The IC50 values of delphinidin for MDA-MB-453 cells and BT474 cells were about 40 and 100 μM, respectively. The phosphorylation of AMPK was enhanced by delphinidin treatment in MDA-MB-453 and BT474 cells. Interestingly, FOXO3, as one of the downstream proteins of AMPK, was observed to be phosphorylated at Ser253. Similarly, Kim et al. reported that paclitaxel could stimulate FOXO3 expression and enhance FOXO3 phosphorylation (Ser253) through an AMPK-dependent mechanism [132]. This study presents the opposite result to the previous study about the anti-cancer effect of casticin and has significance in that the activation of AMPK/FOXO3 signaling can show anti-cancer effects, such as autophagy and apoptosis with a different phosphorylation status of FOXO3.

Shikonin induced apoptosis in lung cancer cells through the activation of FOXO3 [133]. Shikonin treatment (1 to 4 μM) inhibited the growth of non-small-cell lung cancer cells (A549, H358, Calu-6, HCC-2279, NCI-H15, NCI-H1437, NCI-H1703, and NCI-H1229) and induced apoptotic cell death, whereas it exhibited lower cytotoxic effects on normal lung fibroblasts (WI38). Shikonin treatment (1 to 2 mg/kg) induced continuous tumor regression in a subcutaneous xenograft mouse model using A549, H358, and NCI-H1437 cells. The shikonin-induced inactivation of AKT leads to the dephosphorylation of FOXO3 at Ser253, resulting in apoptotic cell death in A549 and NCI-H1437 cells through promoting BIM expression. The regulation of IGFBP1 and FOXO3 revealed a novel mechanism in the ursolic acid-induced inhibition of hepatocellular carcinoma cell growth [134]. Ursolic acid inhibited the proliferation of Bel-7402 hepatocellular carcinoma cells with an IC50 value of about 23 μM. Ursolic acid elevated FOXO3 protein expression by activating p38 MAPK and increasing IGFBP1 expression. The overexpression of IGFBP1 amplified the effect of ursolic acid on FOXO3 expression and the phosphorylation of p38 MAPK. Unfortunately, the specific phosphorylation site in FOXO3 was not investigated in this study. These findings suggest a potential interplay between the tumor suppressors IGFBP1 and FOXO3, forming a feedback regulatory axis that mediates the overall response of ursolic acid in hepatocellular carcinoma cells.

The anti-cancer effect of vernodalin was mediated by FOXO3 in breast cancer cells in vitro and breast tumor growth in vivo [135]. Treatment with vernodalin (17, 26, and 33 μM) for 24 h significantly increased the expression of FOXO3 in both MCF7 and MDA-MB-231 cells, but decreased the phosphorylated FOXO3 at Ser253 compared with the control. Additionally, the levels of p27, p21, and BIM were elevated, while the levels of cyclin D1 and cyclin E were reduced in response to vernodalin treatment. Western blot analysis revealed that FOXO3 expression was higher in the nucleus compared with the cytoplasmic fraction following vernodalin treatment in MCF7 and MDA-MB-231 cells. Flow cytometry analysis showed that silencing FOXO3 with siRNA prevented vernodalin-induced cell death. In untreated LA7 breast tumor-bearing rats, the tumor volume increased to 2143 mm³, whereas a significant reduction in tumor volume was observed in the treatment groups, with volumes measuring 1182 and 524 mm³ for 1 and 10 mg/kg vernodalin injections, respectively.

Berberine triggered apoptosis in lung cancer cells via the p38 MAPK-mediated induction of FOXO3 and p53 [136]. Berberine reduced A549 cell viability with an IC50 value of about 50 μM at 72 h of treatment. The inhibitor of p38 MAPK (SB203580) abolished berberine-induced FOXO3 and p53 protein expression. Similar results were observed with silencing by p38α MAPK siRNAs. Silencing FOXO3 or p53 counteracted the inhibitory effect of berberine on A549 cell growth. These results suggested that the activation of p38α MAPK was involved in the berberine-mediated apoptosis of A549 cells by the induction of FOXO3 and p53 protein expression.

Genistein reduced the proliferation of colon cancer cells by diminishing the negative impact of epidermal growth factor (EGF) on the activity of FOXO3 [137]. The proliferation rate of HT-29 cells by genistein treatment (150 μM) was about 10% of that of the control group under EGF. EGF-induced phosphorylation of FOXO3 at Thr32 was inhibited by genistein, indicating that genistein enhanced FOXO3 activity. The inhibition of FOXO3 phosphorylation by genistein was mediated by the EGF/PI3K/AKT pathway. Genistein prevented EGF-induced translocation, allowing FOXO3 to remain in the nucleus in HT-29 cells. Genistein enhanced FOXO3 binding to the p27 promoter, which increased p27 expression and resulted in cell cycle arrest in HT-29 cells. Resveratrol inhibited the in vitro proliferation of pancreatic cancer cells and in vivo growth of orthotopic pancreatic tumors by activating FOXO3 [138]. Resveratrol reduced cell viability in four kinds of pancreatic cancer cells (MIA PaCa-2, AsPC-1, PANC-1, and Hs766T) in a dose-dependent manner. PANC-1 and MIA PaCa-2 were the most sensitive, AsPC-1 showed moderate sensitivity, and Hs766T was the least sensitive to resveratrol. The IC50 value of resveratrol was observed to range from 20 to 25 μM. The inhibition of AKT and ERK enhanced the apoptosis induced by resveratrol in PANC-1 cells. The overexpression of FOXO3 increased resveratrol-induced apoptosis. Phosphorylation-deficient mutants of FOXO3 had a greater impact on resveratrol-induced apoptosis compared with wild-type FOXO3, suggesting that the phosphorylation of FOXO3 by kinase could play an important role in apoptosis induced by resveratrol in PANC-1 cells.

**Figure 4:**
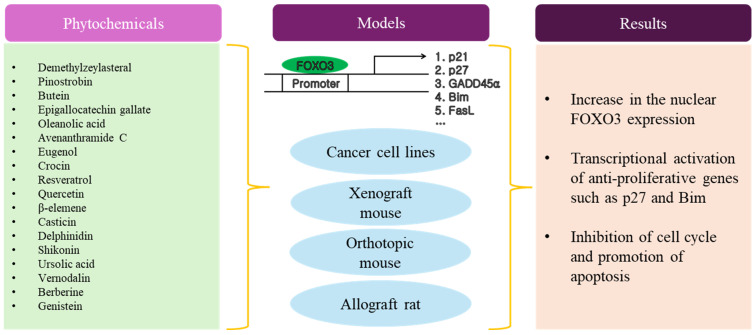
Schematic diagram of anti-aging activity by phytochemicals.

As shown in Table 4 and Figure 4, there have been many reports about the anti-cancer activity of phytochemicals with poly phenol structures, such as flavonoids in fruits and vegetables. Typically, cancer cells were treated with phytochemicals ranging from approximately 10 nM to 3 mM up to 72 h. In this range, the IC50 value of each phytochemical was determined. Interestingly, several studies showed that less toxicity to normal cell lines by each phytochemical was observed, despite treatment at relatively high concentrations of phytochemicals. Most studies used in vitro human cancer cell lines as model systems to investigate the anti-cancer activity of phytochemicals. Regarding the anti-cancer mechanism, most phytochemicals induced cell cycle arrest and apoptosis in cancer cells by activating FOXO3, which led to the transcriptional up-regulation of cell cycle inhibitors (p21 and p27) and pro-apoptotic proteins (BIM, BAX, and FasL). The specific AKT-dependent phosphorylation sites in FOXO3, such as Thr32 and Ser253, were frequently investigated and the dephosphorylation by each phytochemical was shown to be involved in the translocation of FOXO3 from cytosol to the nucleus. The AMPK/FOXO3 signaling pathway is known to have tumor-suppressive properties [139,140,141]. The AMPK/FOXO3 pathway is therefore a critical axis in preventing cancer cell growth and promoting cell death. Interestingly, a few reports demonstrated that the activation of AMPK phosphorylation by specific phytochemicals resulted in a reduction in FOXO3 phosphorylation at Ser253. Both FOXO3 and p53 are critical regulators of cell fate decisions and play significant roles in suppressing tumor formation [142,143]. Overall, while both FOXO3 and p53 have distinct mechanisms of action, they converge on several key pathways to suppress tumor formation, including cell cycle regulation, apoptosis induction, DNA repair, and oxidative stress responses. Recent studies suggest that their coordinated activities by specific phytochemicals contributed significantly to preventing the development of cancer.

While the potential of phytochemicals as anti-cancer agents is promising, it is essential to conduct further research to elucidate their mechanisms of action, optimize dosing regimens, and evaluate their efficacy and safety in clinical settings. Although Phase I and II clinical trials have been completed for only a few selected phytochemicals and their derivatives [144] targeting various cancer types, more robust in vivo animal tests and clinical trials are needed to fully realize their potential as anti-cancer agents.

## 8. Limitations and Future Outlook

Recent studies in the fields of anti-aging and anti-cancer research have made significant progress, yet they still face several limitations. Understanding these limitations and the future directions can help to guide further research and development. First, we need to think about the complexity of aging and cancer mechanisms. Both aging and cancer are influenced by a complex interplay of genetic, environmental, and lifestyle factors [145]. Individual differences in genetics, epigenetics, and metabolism make it difficult to understand all these interactions and develop universal treatments. Secondly, there should be efforts made to transition from preclinical to clinical trials. Many studies just rely on in vitro cell lines or animal models, which may not accurately replicate human aging and cancer processes. There is often a gap between promising preclinical results and successful human trials due to differences in physiology, drug metabolism, and side effects [146]. We also need to pay attention to issues of safety. The long-term safety of anti-aging and anti-cancer treatments is often unknown [147]. Potential side effects, especially for chronic use, need thorough investigation. Some treatments may have toxic effects that limit their use.

Ancient medical practices have long employed herbal remedies to treat various diseases. This ancient wisdom is now being utilized to discover bioactive molecules with potential applications in modern therapy. Phytochemicals from plants used in traditional medicine have been reported to possess the ability to increase longevity and suppress multiple types of cancer. In this review, recent studies suggested that activating FOXO3 through various signaling pathways using natural plant extracts and phytochemicals could be an effective strategy for preventing aging and cancer progress. In order to raise expectations about the anti-aging and anti-cancer effects of phytochemicals, future efforts will be needed to overcome some of the limitations mentioned above. First, we can understand more detailed information on the mechanisms of the aging process and cancer development through cutting-edge systems biology and omics technology [148,149,150,151]. Integrating genomics, proteomics, and metabolomics data can provide a more comprehensive understanding of aging and cancer mechanisms. In addition, further research into epigenetic changes associated with aging and cancer can lead to novel therapeutic targets [152,153,154,155]. Second, we should consider conducting large-scale and well-designed clinical trials to validate the efficacy and safety of new treatments. From robust clinical results, we could tailor treatments to individual people with different genetic information using proper phytochemicals [156,157]. Third, we should develop advanced preclinical model systems, such as humanized animal models and organ-on-a-chip technologies, to better mimic human disease and study the effects of treatments in a more physiologically relevant context [156,158,159]. Fourth, prevention is more important than therapy in managing aging and cancer. Preventive measures through the intake of phytochemicals can significantly slow down aging and lower the incidence of cancer. By addressing risk factors and promoting healthy lifestyle choices, phytochemicals can protect cells and tissues from the aging process and cancer development, diminishing the burden on individuals and healthcare systems [155,160,161].

There is an issue regarding the treatment condition of phytochemicals used in cell culture studies versus nutritionally relevant digestion. Many cell culture studies indeed use pharmacological, rather than nutritional, doses of phytochemicals, which can lead to misleading conclusions about their effects in the human body [162,163,164]. Cell culture studies often use much higher concentrations of phytochemicals than what would typically be achieved through the diet. These pharmacological doses may produce effects that are not representative of what occurs at nutritional levels. The concentrations of phytochemicals that actually reach tissues in the body after ingestion are often much lower than those used in cell culture studies. This is due to factors like poor absorption, metabolism, and rapid excretion. Phytochemicals are often metabolized in the body, resulting in different compounds than those initially present in food or used in cell cultures. These metabolites may have different biological activities. In real dietary situations, phytochemicals are consumed as part of whole foods, where they interact with other compounds. This complexity is often not captured in cell culture studies using isolated compounds. Cell culture studies often involve short-term exposure to high concentrations, whereas dietary intake typically involves long-term exposure to lower concentrations. To address these issues, researchers should use physiologically relevant concentrations based on bioavailability data from human studies. Also, we need to consider the effects of metabolism and use relevant metabolites in cell culture studies. In addition, we need to conduct studies using whole food extracts in addition to isolated compounds and validate cell culture findings with in vivo studies and human clinical trials. It is crucial to interpret cell culture studies with caution and not extrapolate directly to human health effects without supporting evidence from in vivo and clinical studies. While cell culture studies are valuable for understanding mechanisms, their limitations in representing real-world nutritional scenarios must be acknowledged.

Phytochemicals offer a promising avenue for longevity and cancer prevention due to diverse mechanisms of action and potential to work synergistically with conventional therapies. By addressing these limitations and focusing on these future directions, researchers can make significant strides in developing effective anti-aging and anti-cancer therapies that are safe, accessible, and beneficial for a broad population. While research is ongoing, these findings suggest that incorporating phytochemical-rich foods into the diet may play a role in activating FOXO3 and potentially counteracting the aging process.

## 9. Conclusions

Phytochemicals are naturally occurring compounds found in plants and have potential health benefits, including an ability to increase longevity and suppress cancer development. Phytochemicals offer a promising avenue for reducing the aging process and preventing cancer development due to their natural origin, diverse mechanisms of action, and potential to work synergistically with conventional therapies. However, challenges such as variability in potency, bioavailability issues, limited clinical evidence, and potential interactions must be carefully addressed. FOXO transcription factors are a family of proteins that play a role in a variety of cellular processes, including cell growth, differentiation, and apoptosis. The exact mechanisms by which FOXO3 regulates aging and cancer in humans are not fully understood. However, it is thought that FOXO3 plays a role in a variety of processes that are associated with aging and cancer. FOXO3 is a promising target for interventions that aim to slow down the aging process and prevent cancer development. Further research is needed to understand the full role of FOXO3 in aging and cancer to develop safe and effective ways to target FOXO3 for therapeutic purposes. This review is expected to encompass an exploration and discussion of various phytochemicals recognized for their ability to activate FOXO3.

## Figures and Tables

**Table 1:** Plant extracts with anti-aging activity.

No.	Plants	Model	Dose	Mean Lifespan Increase (%)	Mechanism, Experiment	Ref.
1	*Myrciaria trunciflora*	*C. elegans*	250 μg/mL	20	Increase in nFOXO3, Fluorescence microscope	[24]
2	*Holothuria scabra*	*C. elegans*	500 μg/mL	8.12	Increase in nFOXO3, Fluorescence microscope	[26]
3	*Agrimonia procera* Wallr.	*C. elegans*	100 μg/mL	22	Increase in nFOXO3, Fluorescence microscope	[25]
4	*Rubus idaeus* L.	*C. elegans*	80 mg/mL	29.7	Increase in nFOXO3, Confocal microscope	[27]
5	*Glochidion zeylanicum*	*C. elegans*	2.5 μg/mL	48.82	Increase in nFOXO3, Fluorescence microscope	[28]
6	*Moringa oleifera*	*C. elegans*	100 µg/mL	20.4	N/A	[29]
7	*Eugenia caryophyllata* Thunb.	*C. elegans*	1 mg/mL	15.3	Increase in nFOXO3, Fluorescence microscope	[30]
8	*Panax ginseng*	*C. elegans*	1 mg/mL *	23.85	Increase in nFOXO3, Fluorescence microscope	[31]
	*Rehmanniae radix*					
9	*Hibiscus sabdariffa* L.	*C. elegans*	1 mg/mL	24	Increase in nFOXO3, Fluorescence microscope	[32]
10	*Viscum album* var. *coloratum*	*D. melanogaster*	25 μg/mL	5.45 (male)	Increase in nFOXO3, Confocal microscope	[33]
				21.02 (female)		

* Liangyi Gao formula included *Panax ginseng* and *Rehmanniae radix* in 1:2 ratio. N/A: Not available.

**Table 2:** Phytochemicals with anti-aging activity.

No.	Phytochemical	Type	Model	Dose	Mean Lifespan Increase (%)	Mechanism, Experiment	Ref.
1	Dihydromyricetin	Flavonoid	*D. melanogaster*	40 μM	16.07	Increase in nFOXO3, Confocal microscope	[51]
2	Kaempferol	Flavonoid	*C. elegans*	100 μM	10	Increase in nFOXO3, Fluorescence microscope	[52]
	Fisetin	Flavonoid		100 μM	6	Increase in nFOXO3, Fluorescence microscope	
3	Chlorogenic acid	Polyphenol	*C. elegans* (DAF16a)	20 μM	24	Increase in cFOXO3, Confocal microscope	[53]
			*C. elegans* (DAF16f)	20 μM	9		
4	Sulforaphane	Isothiocyanate	*C. elegans*	100 μM	18.2	Increase in nFOXO3, Fluorescence microscope	[54]
5	Phlorizin	Dihydrochalcone	*C. elegans*	10 μM	18	Increase in nFOXO3, Fluorescence microscope	[55]
6	Caffeic acid phenethyl ester	Polyphenol	*C. elegans*	100 μM	9	Increase in nFOXO3, Fluorescence microscope	[56]
7	3-(3,4-dihydroxyphenyl) propionic acid	Phenolic acid	*C. elegans*	0.5 mM	11.2	Increase in nFOXO3, Confocal microscope	[57]
	3-(3-hydroxyphenyl) propionic acid			2.5 mM	13		
	3-phenyl propionic acid			2.5 mM	10.6		
8	Myricetin	Flavonoid	*C. elegans*	100 μM	48.2	Increase in nFOXO3, Fluorescence microscope	[58]
	Laricitrin			100 μM	35.7		
	Syringetin			100 μM	54.5		
	Myricetin trimethyl ether			100 μM	53.6		
9	Indoline carboxylic acid-betacyanin	Betalain	*C. elegans*	10 μM	16.55	Increase in nFOXO3, Fluorescence microscope	[59]
	Phenylalanine-betaxanthin			25 μM	12.92		
	Dopaxanthin			100 μM	20.52		
10	Damaurone D	Aurone	*C. elegans*	15 μM	16.7	Increase in nFOXO3, Fluorescence microscope	[60]
11	Tectochrysin	Flavonoid	*C. elegans*	200 μM	21	Increase in nFOXO3, Fluorescence microscope	[61]
			*C. elegans* (amyloid-β (Aβ1-42))	200 μM	14.8		
12	Syringaresinol	Lignan	*C. elegans*	500 nM	41	Increase in nFOXO3, Confocal microscope	[62]
			*D. melanogaster* (male)	500 nM	10.52		
			*D. melanogaster* (female)	500 nM	13.67		
13	Quercetin	Flavonoid	*C. elegans*	100 μM	15	Increase in nFOXO3, Fluorescence microscope	[63]
14	Quercetin	Flavonoid	Mouse (C57BL/6J, male)	0.125 mg/kg	N/A	N/A	[64]

N/A: Not available.

**Table 3:** Plant extracts with anti-cancer activity.

No.	Plants	Cancer	Cell Line	Dose	Animal Model	Dose, Time	Mechanism, Experiment	Ref.
1	*Houttuynia cordata* Thunb	Liver cancer	HepG2	10 μg/mL	Xenograft mouse	20 mg/kg,	Increase in nFOXO3, Nuclear fractional WB	[82]
					(HepG2)	20 days	Increase in tFOXO3, Total cell lysate WB	
2	*Allium Roseum* L.	Myeloid leukemia	K562	500 µg/mL	N/A	N/A	Decrease in pFOXO3 (N/S), Total cell lysate WB	[83]
3	*Morus alba*	Neuroblastoma	B103 (rat)	10 μg/mL	N/A	N/A	Decrease in pFOXO3 (Thr32), Fluorescence microscope, Total cell lysate WB	[84]
4	*Chelidonium majus*	Ovarian cancer	SKOV3	200 μg/mL	N/A	N/A	Decrease in pFOXO3 (Ser294), Total cell lysate WB	[85]
			OVCAR3	200 μg/mL			Increase in nFOXO3, Nuclear fractional WB	
			MDAH2774	200 μg/mL				
5	*Fagonia cretica*	Breast cancer	MCF7	0.43 mg/mL	N/A	N/A	Increase in tFOXO3, Total cell lysate WB	[86]
			MDA-MB-231	1.01 mg/mL				
6	*Hammada scoparia*	Leukemia	U937	66 μg/mL	N/A	N/A	Increase in pFOXO3 (Thr32), Total cell lysate WB	[87]
			HL60	66 μg/mL				
			KG1	66 μg/mL				
7	*Aegiceras corniculatum*	Colon cancer	HT-29	34.01 μg/mL	Xenograft mouse	25 mg/kg,	Increase in pFOXO3 (Ser253), Total cell lysate WB	[88]
			SW48	37.90 μg/mL	(HT-29)	24 days	Increase in tFOXO3, Total cell lysate WB	
8	*Anagallis arvensis*	Breast cancer	MCF7	33.73 μg/mL	N/A	N/A	Increase in tFOXO3, Total cell lysate WB	[89]
			MDA-MB-231	48.24 μg/mL				

N/A: Not available. N/S: Not specified.

**Table 4:** Phytochemicals with anti-cancer activity.

No.	Phytochemical	Type	Cancer	Cell Line	IC50 (μM) *	Animal Model	Dose, Time	Mechanism, Experiment	Ref.
1	Demethylzeylasteral	Triterpenoid	Lung cancer	H460	8.46	Xenograft mouse	5 mg/kg,	Decrease in pFOXO3 (Thr32), Total cell lysate WB	[114]
				H1975	6.97	(PC-9)	12 days		
				PC-9	8.68				
2	Pinostrobin	Flavonoid	Myeloid leukemia	HL-60	129.9	Xenograft zebrafish	60 μM,	Increase in nFOXO3, Nuclear fractional WB, Confocal microscope	[115]
				U-937	919.7	(MV4-11)	48 h		
				THP-1	1458				
				MV4-11	35.8				
				MOLM-13	53.2				
3	Butein	Chalcone	Ovarian cancer	A2780	64.7	Xenograft mouse	4 mg/kg,	Increase in nFOXO3, Nuclear fractional WB, Increase in tFOXO3, Total cell lysate WB	[116]
				SKOV3	175.3	(A2780)	21 days		
4	Epigallocatechin gallate	Catechin	Ovarian cancer	A2780	0.02	Xenograft mouse	200 mg/kg,	Increase in tFOXO3, Total cell lysate WB	[117]
				SKOV3	0.02	(A2870 or SKOV3)	28 days		
5	Oleanolic acid	Triterpenoid	Colon cancer	HCT116	29.3	Xenograft mouse	10 mg/kg,	Increase in pFOXO3 (Ser294), Total cell lysate WB	[118]
						(HCT116)	33 days	Increase in nFOXO3, Fluorescence microscope	
								Increase in tFOXO3, Total cell lysate WB	
6	Avenanthramide C	Polyphenol	Colon cancer	SW620	153.02	Xenograft mouse	50 mg/kg,	Increase in tFOXO3, Total cell lysate WB	[119]
				SW480	161.47	(HCT-8)	21 days		
				HCT-8	105.23				
				HCT116	112.69				
7	Eugenol	Phenylpropanoid	Breast cancer	MDA-MB-231	20	N/A	N/A	Increase in tFOXO3, Total cell lysate WB	[120]
				SK-BR-3	14				
8	Crocin	Carotenoid	Breast cancer	MCF7	3000	N/A	N/A	Decrease in pFOXO3 (Ser253), Nuclear fractional WB, Total cell lysate WB, Fluorescence microscope Increase in tFOXO3, Total cell lysate WB	[123]
				MDA-MB-231	2700			Increase in nFOXO3, Nuclear fractional WB, Fluorescence microscope	
9	Resveratrol	Polyphenol	Cervical cancer	HeLa	N/A	N/A	N/A	Decrease in pFOXO3 (N/S), Total cell lysate WB	[124]
								Increase in nFOXO3, Confocal microscope	
								Increase in tFOXO3, Total cell lysate WB	
10	Quercetin	Flavonoid	Cervical cancer	HeLa	100	N/A	N/A	Increase in FOXO3 mRNA, Real-time PCR	[125]
11	β-elemene	Sesquiterpene	Lung cancer	H1957	30	Xenograft mouse	75 mg/kg,	Increase in tFOXO3, Total cell lysate WB	[126]
				A549	N/A	(A549)	16 days		
12	Casticin	Flavonoid	Lung cancer	H446	3	N/A	N/A	Decrease in pFOXO3 (Ser253), Total cell lysate WB	[129]
13	Delphinidin	Anthocyanidin	Breast cancer	MDA-MB-453	40	N/A	N/A	Increase in pFOXO3 (Ser253), Total cell lysate WB	[131]
				BT474	100				
14	Shikonin	Naphthoquinone	Lung cancer	A549	1	Xenograft mouse	1 or 2 mg/kg,	Decrease in pFOXO3 (Ser253), Total cell lysate WB Increase in nFOXO3, Fluorescence microscope, Nuclear fractional WB	[133]
				H1437	1	(A549, H1437, or	8 or 15 days		
				Calu-6	1	Calu-6)			
15	Ursolic acid	Triterpenoid	Liver cancer	Bel-7402	23	Xenograft mouse	50 mg/kg,	Increase in tFOXO3, Total cell lysate WB	[134]
				HepG2	23	(HepG2)	30 days		
16	Vernodalin	Sesquiterpene	Breast cancer	MCF7	N/A	Allograft rat	10 mg/kg,	Decrease in pFOXO3 (Ser253), Total cell lysate WB	[135]
				MDA-MB-231	N/A	(LA7)	35 days	Increase in nFOXO3, Nuclear fractional WB	
								Increase in tFOXO3, Total cell lysate WB	
17	Berberine	Alkaloid	Lung cancer	A549	50	N/A	N/A	Increase in tFOXO3, Total cell lysate WB	[136]
18	Genistein	Isoflavone	Colon cancer	HT-29	100	N/A	N/A	Decrease in pFOXO3 (Thr32), Total cell lysate WB Increase in nFOXO3, Confocal Microscope	[137]
19	Resveratrol	Polyphenol	Pancreatic cancer	MIA PaCa-2	20	Orthotopic mouse	60 mg/kg,	Decrease in pFOXO3 (Ser253), Total cell lysate WB	[138]
				AsPC-1	25	(PANC-1)	42 days		
				PANC-1	25				
				Hs766T	25				

*: IC50 value based on cell proliferation assays, such as MTT and cell-counting assay. pFOXO3: Phosphorylated FOXO3. nFOXO3: Nuclear FOXO3. cFOXO3: Cytosolic FOXO3. tFOXO3: Total FOXO3. N/A: Not available. N/S: Not specified.

## Data Availability

The author declares that there are no primary datasets and computer codes associated with this study. All data and materials are available to the researchers once published.
